# Oxygen uptake efficiency slope as a useful measure of cardiorespiratory fitness in morbidly obese women

**DOI:** 10.1371/journal.pone.0172894

**Published:** 2017-04-06

**Authors:** Tatiana Onofre, Nicole Oliver, Renata Carlos, Amanda Felismino, Renata Cristina Corte, Eliane Silva, Selma Bruno

**Affiliations:** 1 Cardiopulmonary and Metabolic Rehabilitation Laboratory, Postgraduate Physical Therapy Program, Federal University of Rio Grande do Norte, Natal, Rio Grande do Norte, Brazil; 2 Integrated Medicine Department, Federal University of Rio Grande do Norte, Natal, Rio Grande do Norte, Brazil; 3 Cardiopulmonary and Metabolic Rehabilitation Laboratory, Physical Therapy Department, Federal University of Rio Grande do Norte, Natal, Rio Grande do Norte, Brazil; Nanyang Technological University, SINGAPORE

## Abstract

Cardiopulmonary assessment through oxygen uptake efficiency slope (OUES) data has shown encouraging results, revealing that we can obtain important clinical information about functional status. Until now, the use of OUES has not been established as a measure of cardiorespiratory capacity in an obese adult population, only in cardiac and pulmonary diseases or pediatric patients. The aim of this study was to characterize submaximal and maximal levels of OUES in a sample of morbidly obese women and analyze its relationship with traditional measures of cardiorespiratory fitness, anthropometry and pulmonary function. Thirty-three morbidly obese women (age 39.1 ± 9.2 years) performed Cardiopulmonary Exercise Testing (CPX) on a treadmill using the ramp protocol. In addition, anthropometric measurements and pulmonary function were also evaluated. Maximal and submaximal OUES were measured, being calculated from data obtained in the first 50% (OUES_50%_) and 75% (OUES_75%_) of total CPX duration. In one-way ANOVA analysis, OUES did not significantly differ between the three different exercise intensities, as observed through a Bland-Altman concordance of 58.9 mL/min/log(L/min) between OUES_75%_ and OUES_100%_, and 0.49 mL/kg/min/log(l/min) between OUES/kg_75%_ and OUES/kg_100%_. A strong positive correlation between the maximal (r = 0.79) and submaximal (r = 0.81) OUES/kg with oxygen consumption at peak exercise (VO_2peak_) and ventilatory anaerobic threshold (VO_2VAT_) was observed, and a moderate negative correlation with hip circumference (r = -0.46) and body adiposity index (r = -0.50) was also verified. There was no significant difference between maximal and submaximal OUES, showing strong correlations with each other and oxygen consumption (peak and VAT). These results indicate that OUES can be a useful parameter which could be used as a cardiopulmonary fitness index in subjects with severe limitations to perform CPX, as for morbidly obese women.

## Introduction

Cardiopulmonary Exercise Testing (CPX) has been widely applied to assess the cardiopulmonary fitness of morbidly obese individuals [1–3]. Similar to other clinical incapacitating conditions, the obese are more susceptible to develop several alterations and diminished response during CPX [4,5]. The main finding is reduced oxygen consumption relative to body weight at exercise peak (VO_2peak_, mL/kg/min) when compared to lean individuals [6,7]. This low capacity for exercise can be influenced by cardiovascular, pulmonary or peripheral reasons [8,9]. Furthermore, individuals with extreme obesity can present energy inefficiencies and biomechanical abnormalities, mainly due to alterated walking pattern and/or orthopedic pain which can limit their maximal physical effort [10,11]; in turn, it contributes to abnormal response with early interruption of CPX [5].

In addition to the primary clinically and scientifically accepted key variables, other additional variables based on emerging scientific evidence have been gaining acceptance in cardiorespiratory assessment during CPX. Considering this, oxygen uptake efficiency slope (OUES) was first proposed by Baba et al. [12] as a submaximal index of cardiopulmonary functional reserve. OUES is derived from the relationship between oxygen consumption (VO_2_, mL/min; plotted on the y axis) and the log transformation of minute ventilation (VE, L/min; x axis). Thus, it is a metric that expresses the ventilatory requirement for a given VO_2_. OUES has been extensively assessed and correlated with VO_2peak_ in cardiac diseases [13–15], indicating it as a valid index for exercise tolerance. A consistent body of literature [14,16–18] has demonstrated that exercise intensity (submaximal or maximal) does not affect OUES values, thus being a reason for the expressive use of this index in clinical conditions which prevent patients from being able to perform an entire CPX.

OUES has been extensively applied in heart [13,14,19] or pulmonary disease [18,20,21], or both [22], and has revealed significantly lower OUES according to the disease severity. Reference equations for OUES have been proposed [23,24], however until now the use of OUES has not been established as a measure of cardiorespiratory fitness in an obese adult population. Only two studies have been conducted with OUES in obese, albeit using a pediatric sample [25,26]. The cardiopulmonary condition through OUES data have demonstrated clinical promise, revealing that we can obtain important clinical information using OUES about functional status and disease severity without necessarily exposing the patient to maximal physical effort [14,16,27].

Thus, the main aim of this study was to characterize submaximal and maximal OUES in morbidly obese women; secondly, to analyse the relationship among OUES and the traditional measures of cardiorespiratory fitness, anthropometry and pulmonary function, and lastly to understand if these results produce the same clinical information in other populations. Taking into account that morbidly obese individuals have extreme physical limitations and cannot sustain exercise at maximal level and due to a lack of data in this specific population, we hypothesized that OUES can be potentially useful to assess functional capacity in these subjects.

## Materials and methods

### Subjects and procedures

All participants signed an informed consent before start the research procedure. With Human Research Ethics Committee approval from the Federal University of Rio Grande do Norte-UFRN (488.283), signature of free informed consent form and trial registration number (RBR-7m2756) in ReBEC, we prospectively assessed the anthropometry, pulmonary function and aerobic capacity as a preoperative routine of bariatric surgery in the Cardiopulmonary Rehabilitation and Metabolic Unit of University Hospital-UH/UFRN. Thirty-three morbidly obese women (mean±SD age of 39.1±9.2years; weight 117.7±16.6kg; height 158.1±6.2cm) were selected by convenience from Obesity Surgery and Related Disease Service of the UH and met the inclusion criteria of the study, being: adult females (age range between 20 and 59 years), and having body mass index (BMI) ≥ 40kg/m^2^. Women with orthopedic limitations, chronic renal failure requiring dialysis, diagnosed pulmonary or cardiovascular diseases were excluded.

### Outcome measures

Anthropometric measures of general (weight, height) and peripheral adiposity (neck—NC, waist—WC, and hip—HC circumferences) were taken to calculate BMI, body adiposity index (BAI) [28] and waist-to-hip ratio (WHR). Spirometric function [29] was assessed according to acceptability and reproducibility of the American Thoracic Society (ATS) [30], using a previously calibrated DATOSPIR 120 C spirometer (SIBELMED, Barcelona, Spain W20).

**Fig 1:**
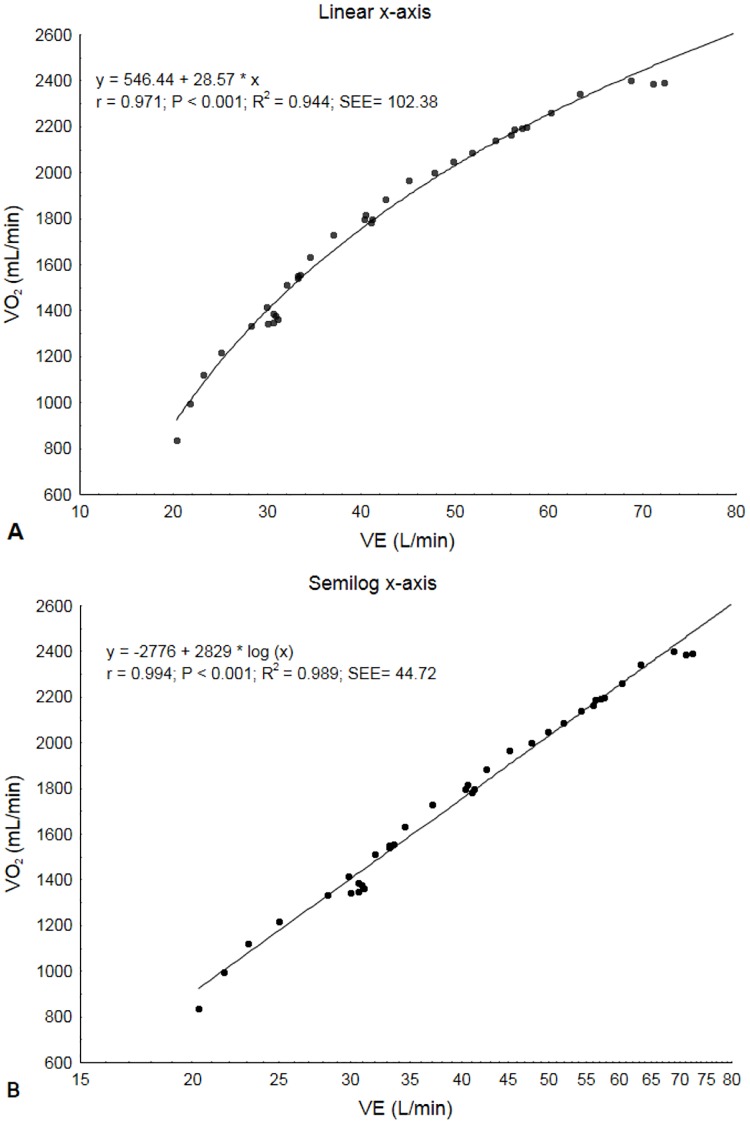
The relationship between VO_2_ and VE during cardiopulmonary exercise testing in a single 36-year old morbidly obese woman. A, Linear x-axis. B, Semilog x-axis (OUES). VO_2_, oxygen consumption; VE, minute ventilation; OUES, oxygen uptake efficiency slope; SEE, standard error.

Symptom-limited CPX was performed on a treadmill (Centurion 300, Micromed) using a breath-by-breath gas analyzer (Cortex Metamax3B Biophysik, Germany). Ramp protocol [31] up to the tolerable limit for the patient was implemented and the subjects were continuously monitored by electrocardiographic 12-lead tracing (ErgoPC Elite 3.4). Blood pressure measurements, peripheral O_2_ saturation and perceived exertion using the Borg Scale were recorded every 2 minutes. Metabolic/ventilatory variables (VO_2_; carbon dioxide production, VCO_2_ and VE-minute ventilation) were measured continuously. Derived variables such as respiratory exchange ratio (RER) and ventilatory equivalent for oxygen (VE/VO_2_) and carbon dioxide (VE/VCO_2_) were also analyzed, as well as VE/VCO_2slope_ and partial pressures of expired oxygen (PetO_2_) and carbon dioxide (PetCO_2_). VO_2peak_ was expressed as the highest VO_2_ reached during the last 10 seconds of the test. Ventilatory anaerobic threshold (VAT) was determined through VE/VO_2_ and VE/VCO_2_ curves, considering the beginning of the turning point boundary in the VE/VO_2_ graph, as the VE/VCO_2_ remained constant, or by using the V-slope method [32]. Each test was preceded by daily calibration of the apparatus. The criteria for CPX interruption were strictly followed in accordance with the standardization of ATS [33]. We used the equations proposed by Wasserman et al. [34] to analyze the predicted VO_2peak_ values. OUES resulted from linear regression analysis from the following equation:

$${\text{VO}}_{\text{2}}={\text{ a x log}}_{\text{10}}\text{VE}+\text{b}$$

which the constant "a" is the value of OUES, which represents the rate of increase in VO_2_ in response to a given increase in VE, and "b" is the intercept (Fig 1). In order to assess its usefulness as a cardiopulmonary index derived from a submaximal exercise test, OUES was also calculated from data obtained in the first 50% (OUES_50%_) and 75% (OUES_75%_) of the total CPX duration. The prediction equation used for OUES values was recently proposed by Buys et al. [35]. As body weight directly affects the relative VO_2_ values reached, and OUES results from a regression analysis involving VO_2_, we also calculated OUES relative to body weight (OUES/kg).

### Statistical analysis

Were estimated power and sample size based on effect magnitude among OUES/mL 100% and 75%. It was necessary 36 women to detect 30.6mL of difference considering α (two sided, 0,05%) and β (error type I) of 20%.

Unless otherwise specified, the data were presented as mean and standard deviation. Pearson correlation coefficients were calculated to test the possibility and magnitude of association between the three intensities of OUES (50%, 75%, 100%) and CPX respiratory/metabolic parameters, anthropometric measurements and pulmonary function. Linear regression analysis was used to investigate the relationships of OUES values with each other and the variables that were significant in the initial correlation model, meaning as a variable influencing the other. In order to verify the existence of significant differences between the OUES means and the three different levels of exercise (factor), an analysis of variance (one-way ANOVA) was performed. ANCOVA was performed to test effect of age and WHR as potencial confounding on final OUES. Bland-Altman plot (SPSS for Windows, Version 20.0, Chicago, IL, USA) was used to assess the level of agreement between maximal and submaximal OUES (including confidence interval-CI) and other analyzes were performed using the Statistica version 10.0 software (StatSoft, USA). A level of P<0.05 was considered to be statistically significant.

## Results

In total, 45 morbidly obese women were recruited; however, 12 were excluded for the following reasons: chronic renal failure requiring dialysis, n = 2; uncontrolled arrhythmias, n = 2; orthopedic impairments, n = 5; and asthma, n = 3. Thus, 33 patients remained (mean±SD age = 39.1±9.2years; height = 158.1±6.2cm; BMI = 47.1±5.8kg/m^2^), with their clinical and anthropometric characteristics and pulmomary function shown in Table 1. Self-reported dyspnea was the main cause of interrupting CPX, corresponding to 60.6% of the sample, followed by fatigue of the lower limbs (30.3%), maximum heart rate-HRmax>predicted (6.1%) and systolic blood pressure>220mmHg associated with hemodynamic repercussions (3.0%).

**Table 1:** Clinical and anthropometric characteristics and pulmonary function of study population.

	Morbidly Obese Women (n = 33)
Variables	
Age (years)	39.1 ± 9.2
Hypertension	20 (60.6%)
Type 2 Diabetes	8 (24.2%)
Ex-smoker	9 (27.3%)
Weight (kg)	117.7 ± 16.6
Height (cm)	158.1 ± 6.2
BMI (kg/m^2^)	47.1 ± 5.8
NC (cm)	39.1 ± 2.9
WC (cm)	125.6 ± 11.2
HC (cm)	140.7 ± 13.1
BAI (%)	52.9 ± 7.1
WHR	0.89 ± 0.08
BSA (m^2^)	2.13 ± 0.16
FVC (L)	2.88 ± 0.46
%predicted FVC	85.2 ± 8.9
FEV_1_ (L/)	2.50 ± 0.45
%predicted FEV_1_	88.7 ± 10.6
FEV_1_/FVC	0.86 ± 0.06
MVV (L/min)	94.1 ± 13.8
%predicted MVV	79.7 ± 10.3

Values expressed as mean ± SD or n (%). BMI, body mass index; NC, neck circumference; WC, waist circumference; HC, hip circumference; BAI, body adiposity index; WHR, waist-hip ratio; BSA, body surface area; FVC, forced vital capacity; FEV_1_, forced expiratory volume in 1 s; MVV, maximal voluntary ventilation.

**Fig 2:**
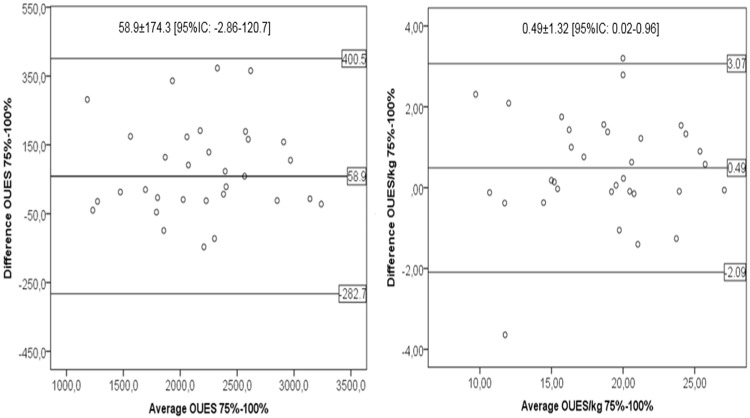
Bland-Altman plots of the relationship of OUES_75%_ and OUES_100%_. Each dot corresponds to a subject. Horizontal lines represent the bias and the upper and lower limits of agreement.

The cardiorespiratory parameters including the maximal and submaximal OUES values during CPX are shown in Table 2. Using one-way ANOVA and ANCOVA analysis, we observed there was no significant difference in mean OUES values (P = 0.420) or OUES/kg (P = 0.430) between three different exercise intensities (50%, 75%, 100%). ANCOVA do not showed effect on OUES/kg by age (P = 0.876), and WHR (P = 0.960). Using Bland-Altman plotting, we also observed an agreement of 58.9 mL/min /log(L/min) (95%; CI: -282.7–400.5) between OUES_75%_ and OUES_100%_, and of 0.49 mL/kg/min/log(L/min) (95%; CI: -2.09–3.07) between OUES/kg_75%_ and OUES/kg_100%_ (Fig 2).

**Table 2:** Distribution of cardiorespiratory parameters during CPX in 33 morbidly obese women.

	CPX			
Variables	VAT		Peak	
VO_2_ (mL/min/kg)	15.2 ± 3.3		18.5 ± 3.6	
%predicted VO_2peak_	61.6 ± 13.2		75.5 ± 14.1	
VO_2_ (mL/min)	1764.2 ± 387.1		2145.7 ± 420.1	
VE (L/min)	43.2 ± 9.6		67.2 ± 14.3	
RER	0.86 ± 0.05		1.03 ± 0.15	
VE/VO_2_	23.3 ± 2.5		30.2 ± 6.3	
VE/VCO_2_	26.7 ± 1.7		29.2 ± 3.2	
PetO_2_ (mmHg)	101.3 ± 4.8		110.1 ± 7.0	
PetCO_2_ (mmHg)	40.9 ± 2.8		37.9 ± 4.0	
HR_max_ (bpm)	148.2 ± 15.6		169.9 ± 17.6	
%predicted HR_max_	81.7 ± 6.9		93.8 ± 7.2	
Total duration (min)	-		6.8 ± 1.8	
VAT time (min)	4.7 ± 1.7		-	
	**OUES**			
	**50%**	**75%**	**100%**	**P value**^a^
OUES (mL/min/log(L/min))	2328.7 ± 633.5	2205.1 ± 547.6	2146.2 ± 530.2	0.420
%predicted OUES	81.6 ± 20.0	77.5 ± 18.5	75.5 ± 18.5	0.420
OUES/kg (mL/kg/min/log(L/min)	19.9 ± 4.9	18.9 ± 4.6	18.4 ± 4.6	0.430

Values expressed as mean ± SD. CPX, cardiopulmonary exercise testing; VAT, ventilatory anaerobic threshold; VO_2_, oxygen consumption; VE, minute ventilation; RER, respiratory exchange ratio; VE/VO_2_, ventilatory equivalent ratio for oxygen; VE/VCO_2_, ventilatory equivalent ratio for carbon dioxide; PetO_2_, end-tidal partial pressure of oxygen; PetCO_2_, end-tidal partial pressure of carbon dioxide; HR, heart rate; OUES, oxygen uptake efficiency slope.

^a^ P values were calculated using One-way ANOVA.

**Fig 3:**
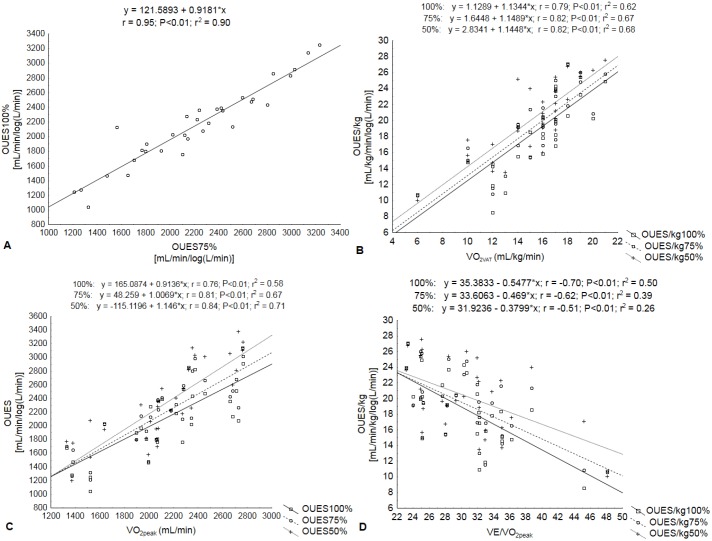
Simple linear regression equation and correlation coefficient showing the relationship between OUES (50%, 75%, 100%) and cardiorespiratory variables during CPX in morbidly obese women. A, OUES_100%_-OUES_75%_. B, OUES/kg_50%_,_75%_,_100%_-VO_2VAT_. C, OUES_50%_,_75%,100%_-VO_2peak_. D, OUES/kg_50%,75%,100%_-VE/VO_2peak_. OUES, oxygen uptake efficiency slope; VO_2VAT_, oxygen consumption at ventilatory anaerobic threshold; VO_2peak_, oxygen consumption at peak exercise; VE/VO_2peak_, ventilatory equivalent ratio for oxygen at peak exercise.

Weak correlations were found between OUES_100%_ and anthropometric measures, however, when adjusted for body weight, OUES/kg_100%_ moderately correlated with HC (r = -0.46, P<0.05) and BAI (r = -0.50, P<0.01). There were no significant correlations between OUES values with pulmonary function (FEV_1_ and FVC), only a weak correlation between OUES_75%_ and MVV (r = 0.39, P<0.05). However, maximal and submaximal OUES were strongly correlated with VO_2peak_, VO_2VAT_ and predicted% OUES values, while being moderately correlated with VE/VO_2peak_ (Table 3). Some of these relationships may be seen in Fig 3, also showing (through equations generated by simple linear regression analysis) the variability between cardiorespiratory parameters in the three OUES intensities, as well as a strong positive correlation between OUES_100%_ and OUES_75%_ (r = 0.95, P <0.01).

**Table 3:** Pearson correlation coefficients showing the relationship between OUES and anthropometric variables and CPX parameters.

	OUES			OUES/kg		
Variables	50%	75%	100%	50%	75%	100%
Age	-0.43*	-0.39*	-0.33	-0.28	-0.20	-0.12
Weight	0.53**	0.36*	0.27	-0.19	-0.29	-0.30
Height	0.46*	0.48**	0.36	0.34	0.31	0.17
HC	-0.03	-0.16	-0.23	-0.25	-0.39*	-0.46*
BAI	-0.28	-0.41*	-0.40*	-0.41*	-0.52**	-0.50**
VO_2peak_ (mL/kg/min)	0.61**	0.70**	0.73**	0.79**	0.81**	0.79**
VO_2peak_ (mL/min)	0.84**	0.82**	0.76**	0.77**	0.67**	0.58**
VO_2VAT_ (mL/kg/min)	0.69**	0.76**	0.77**	0.82**	0.81**	0.79**
VO_2VAT_ (mL/min)	0.84**	0.83**	0.77**	0.75**	0.66**	0.58**
VE/VO_2peak_	-0.38*	-0.54**	-0.66**	-0.51**	-0.62**	-0.70**
%predicted OUES	0.57**	0.81**	0.91**	0.76**	0.86**	0.97**

OUES, oxygen uptake efficiency slope; CPX, cardiopulmonary exercise testing; HC, hip circumference; BAI, body adiposity index; MVV, maximal voluntary ventilation; VO_2_, oxygen consumption; VAT, ventilatory anaerobic threshold; VE/VO_2_, ventilatory equivalent ratio for oxygen.

*P<0.05

**P<0.01.

## Discussion

This is the first study that has investigated the usefulness of OUES in evaluating maximal and submaximal physical effort in morbidly obese women. We tested OUES at 50%, 75% and in the complete test (100%), and its relationship among metabolic, ventilatory and anthropometric variables. The main finding shows that the OUES values did not significantly change independent of exercise intensity, showing strong correlation between OUES (maximal and submaximal) and VO_2peak_. On the other hand, both VO_2peak_ as well as OUES_100%_ decreased, representing 75.5% of predicted values.

Although VO_2peak_ is considered the best standard to measure aerobic fitness for several health or clinical conditions, some limited patients such as those with heart failure, multiple sclerosis or being morbidly obese could have a limited VO_2peak_ from being in such poor physical condition, having impaired pulmonary function, skeletal muscle injuries or having a very heavy body. Due to these conditions, medical illnesses induce a low exercise capacity and VO_2peak_ cannot be accurately assessed. From the clinical exercise and sports medicine perspective, other submaximal exercise variables could be considered in morbidly obese patients; for example, using OUES as a functional reserve index. Similar to other clinical conditions [14,15,36], or even considering three different exercise intensities, we have shown a low influence of intensity on OUES outcome. When we extrapolate the analysis for the submaximal and maximal levels of OUES, we notice a strong positive correlation between VO2_peak_ and OUES in the complete test and for the 50% and 75% tests (Fig 3C). This fit occurs even when OUES was normalized for body weight and did not differ among the three exercise intensities. Also, there was only 2.7% variance between OUES_75%_ and OUES_100%_. Furthermore, the Band-Altman plot showed good agreement between them with a difference of only 58.9 mL/min/log(L/min) for OUES and 0.49 mL/kg/min/log(L/min) for OUES/kg. Our results confirm the previous findings of Laethem et al. [14] who found a 3.0% difference using the same intensity of our OUES in heart failure. Initially, these findings were also reproducible in 429 healthy adult subjects [16]. Bongers et al. [18] found similar results (4.3%) for children with cystic fibrosis using the same OUES intensity as in our study. One of the important aspects to consider is that even though Bland–Altman analysis is designed to overcome some of the limitations of the correlation approach for the purpose of agreement assessment, we don’t know if 0.49 or 58.9 are acceptable clinical limits to attribute as being good agreement. Until now there has not been any data for morbidly obese patients.

Our study corroborates many previous studies [15,18,26] regarding the strong correlation between the maximal (r = 0.76) and submaximal OUES (r = 0.82) with VO_2peak_. In obese children [26], OUES_100%_ and OUES_VAT_ were correlated with VO_2peak_ (L/min) as 0.91 and 0.71, respectively. For heart congenital disease [15], similar results of 0.89 were found for OUES maximal and 0.71 at 50%, also related to VO_2peak_. In our data, both OUES (r = 0.77) and OUES/kg (r = 0.79) were also strongly correlated with VO_2VAT_. From the physiological perspective, VAT is used as a submaximal estimate of aerobic potential; however, in severe obesity, this event seems to be difficult to reach. OUES is more easily detected than VTA, and unlike VO_2peak_, it is less influenced by motivational patient factors which affects performance in CPX [14,21]. This is a point where OUES has a huge advantage because it is independent of exercise intensity and the patient's motivation, which allows for it to be used as a cardiorespiratory functional reserve index.

The maximal level of effort should be certified by RER ≥ 1.10 during CPX [37]. Our data for the mean effort at peak exercise was 1.03, and 75.8% did not reach this level. This differed from de DeJong et al. [4], where out of 76 morbidly obese (69.3% female), only 43.0% did not reach RER ≥ 1.10 at the end of CPX. Miller et al. [38] found that 33% of 64 morbidly obese (78.0% female) did not reach RER ≥ 1.10. Two possible reasons could explain these findings. First, data from men could raise the RER, since they tend to have more fitness than women [39]. Second, the type of protocol implemented was different from our ramp protocol, where those authors used the modified Bruce protocol which increments work every three minutes, allowing the respiratory, cardiac and musculoskeletal systems more time to adapt and avoids the early interruption of the test. However, more studies are needed to better assess this theory.

Several studies [14,21,26] in other populations have shown that OUES and OUES/kg are divergent from ours; this shows the importance of studying OUES in the obese population. For example, the women in our study had 21.3% less OUES than obese girls shown by Breithaupt et al. [26]. Two large studies involving prediction equations for OUES [23,24] showed that age is a factor that can negatively influence OUES values. However, as our sample did not have large variations in age, we could not observe a significant relationship between OUES and age. The obese subjects in our study had 22.2% more OUES than patients with cardiac sarcoidosis, as shown by Ammenwerth et al. [21]. Several aspects should be considered for assesssing OUES. Higher values of OUES depend on adequate heart rate, oxygen extraction and utilization by peripheral muscles, and later lactic acidosis [14,16]. It is known that ventilation of physiological dead space which depends on the structural integrity of lungs and adequate lung perfusion can influence the ventilatory response to exercise, and consequently the values of OUES [16]. A maximal effort by the heart, respiratory and musculoskeletal system is necessary. However, individuals in poor physical condition (such as morbidly obese) or patients who are affected by some chronic illness have a high probability to develop early lactic acidosis during exercise [32]. In this way they became unable to maintain the CPX at a maximal level, thus presenting reduced VO_2peak_ values, and consequently lower OUES than predicted.

As a second objective, we were able to establish a negative influence of OUES on BAI (r = -0.40) and OUES/kg on BAI (r = -0.50) and HC (r = -0.46). In other words, the higher the body fat/adiposity index and hip circumference, the lower the OUES of our sample, since it was an extremely obese population (BMI = 47.1) with high hip circumference values (HC = 140.7) and indirectly calculated fat percentage (BAI = 52.9%). As defined by Bergman et al. [29], BAI is an alternative measurement which is easy to apply and useful to quantify the percentage of body fat. It showed a positive correlation (0.85) with Dual-energy X-Ray Absorptiometry (DXA), being a gold standard for this assessment [29]. The vast amount of fat around the hips is believed to hinder the walking ability of obese and is associated with higher perception of effort, leading to early exhaustion [40,41]. This reduced exercise tolerance is reflected by low values of VO_2peak_ (mL/kg/min) during CPX, thereby affecting OUES.

Until now, little has been studied on the relationship between OUES and VE/VO_2_. Only one study [42] in heart failure disease showed moderate correlation (r = -0.58) between these variables. Our findings demonstrate higher values to explain this relationship, where the VE/VO_2_ correlated negatively with OUES and OUES/kg at -0.66 and -0.70, respectively. Different from heart failure, severe obesity causes significant inefficiency in respiratory and body mechanics caused by excess body fat around the thorax and abdomen, being responsible for increasing respiratory work and pulmonary ventilation [43,44]. In addition, a high demand of ventilation during exercise in obese individuals will reflect an increase in metabolic expenditure and inadequate ventilatory reserves [45,46]. Taken together, these alterations could justify the strong negative correlation between OUES and VE/VO_2_, where it seems that OUES is a variable obtained by the inclination curve of VE and VO_2_, and could be considered a marker of ventilatory efficiency.

The following limitations should be considered in our study. First, a relatively small and homogeneous sample makes it difficult to extrapolate these results to other obesity profiles, but the inclusion of only morbidly obese women removes bias. If we had included men, the sample could be contaminated due to their greater aerobic capacity, and we chose obese women with high BMI because it is generally not possible to evaluate their maximum effort. Another strong aspect of our data is that we included sedentary women with healthy pulmonary function and reasonable ventilatory endurance, which ensured that respiratory function was minimally preserved during CPX. We also recognize that we did not evaluate the reproducibility of OUES. However, OUES has previously seemed to be well reproducible in pediatric populations [12] and healthy adults [47,48]. Finally, given the huge lack of studies with OUES in the morbidly obese, our primary intention was to improve the physiological understanding and typical profile of OUES, and because of this we did not include a control group.

## Conclusions

Taking into account the results and limitations of our study, we conclude that OUES is a favorable parameter that can be applied to morbidly obese women without difference between maximal (OUES_100%_) and submaximal (OUES_50%_, OUES_75%_) levels, showing a strong positive correlation with measures of physical functioning (VO_2peak_ and VO_2VAT_). We suggest that OUES is a useful index of cardiorespiratory fitness and could be used as an alternative for aerobic capacity by providing an objective measure of cardiopulmonary function, mainly in evaluating individuals with limitations or incapacity to perform maximal physical effort during CPX, such as in the case of morbidly obese women. However, this does not take away the need for complete CPX when there are other mandatory clinical reasons or to prescribe exercise.

## Supporting information

S1 File Minimal data. (XLSX)
